# After egg collection, can we predict the chance of embryos for day 5 transfer or freezing?

**DOI:** 10.1530/RAF-21-0018

**Published:** 2021-09-09

**Authors:** I Robertson, F P Chmiel, Y Cheong

**Affiliations:** 1 Human Development and Health, University of Southampton, Southampton, UK; 2 IT Innovation Centre, School of Electronics and Computer Science, University of Southampton, Southampton, UK; 3 Human Development and Health, University of Southampton, Southampton, UK; 4 Complete Fertility, Princess Anne Hospital, Southampton, UK

## Abstract

Even partway through an IVF cycle, at the point when a woman’s eggs have been collected, it is hard to provide reliable answers to the common question of ‘Am I likely to have a good embryo to transfer?’ Sometimes, it only takes one good egg to be successful. However, doctors and patients are acutely aware that low egg numbers, older age and having conditions such as endometriosis can stack the odds against success. We have developed a model to try and answer this question for those patients who wish for more information to help guide their expectations after egg collection. A new tool is presented to predict whether a woman having IVF treatment will have a good enough embryo either to transfer on day 5 or freeze. It was built using information from all 2015 to 2016 UK cycles and predicts using age, number of eggs collected and cause of subfertility.

## Introduction

The outcome of* in vitro* fertilisation (IVF) is prognosticated in terms of ‘live births’, a key outcome only measurable at the end of pregnancy after treatment. In reality, clinicians are required to inform patients of progress throughout the entire treatment journey. However, even after egg collection, clinicians cannot accurately predict the patients’ likelihood of obtaining ≥1 suitable embryos(s) for day 5 (D5) transfer or cryopreservation. This work aims to develop a reliable model and simple tool to improve this prediction and facilitate communication.

## Methods

Retrospective analysis developing and validating a machine-learning model using the HFEA 2015–2016 anonymised register (HFEA 2018). In total, 86,169 IVF/ICSI cycles met the specified inclusion criteria (IVF/ICSI cycles for treatment now, using own oocytes and partner or donor sperm, not PGD/PGS or surrogacy). The outcome of interest was whether the cycle resulted in a day 5 embryo transferred or at least one D5 embryo(s) frozen for future use. We included ‘or freezing’ to incorporate freeze-all cycles where D5 embryos were created and stored in the positive outcome group.

After cohort selection, 20% of treatment cycles (stratified by age group) were used as a hold-out test set. Model training was performed using the remaining treatment cycles using five-fold cross-validation. The sci-kit learn Python library was used to implement this training and validation procedure (Pedregosa *et al.* 2011). As a classifier, we use the XGBoost libraries implementation of a gradient-boosted decision tree because of its high performance and ability to handle missing data natively (Chen *et al.* 2010). Models were constructed for each individual variable and for selected subsets for comparison. For the final model, hyperparameter tuning was performed using the out-of-fold samples in the cross-validation process. The highest-performing model was finally evaluated on the hold-out test set to provide a robust test of the model’s generalization to new samples. Model performance is presented as AUROC with associated CIs.

## Results

**Figure 1:**
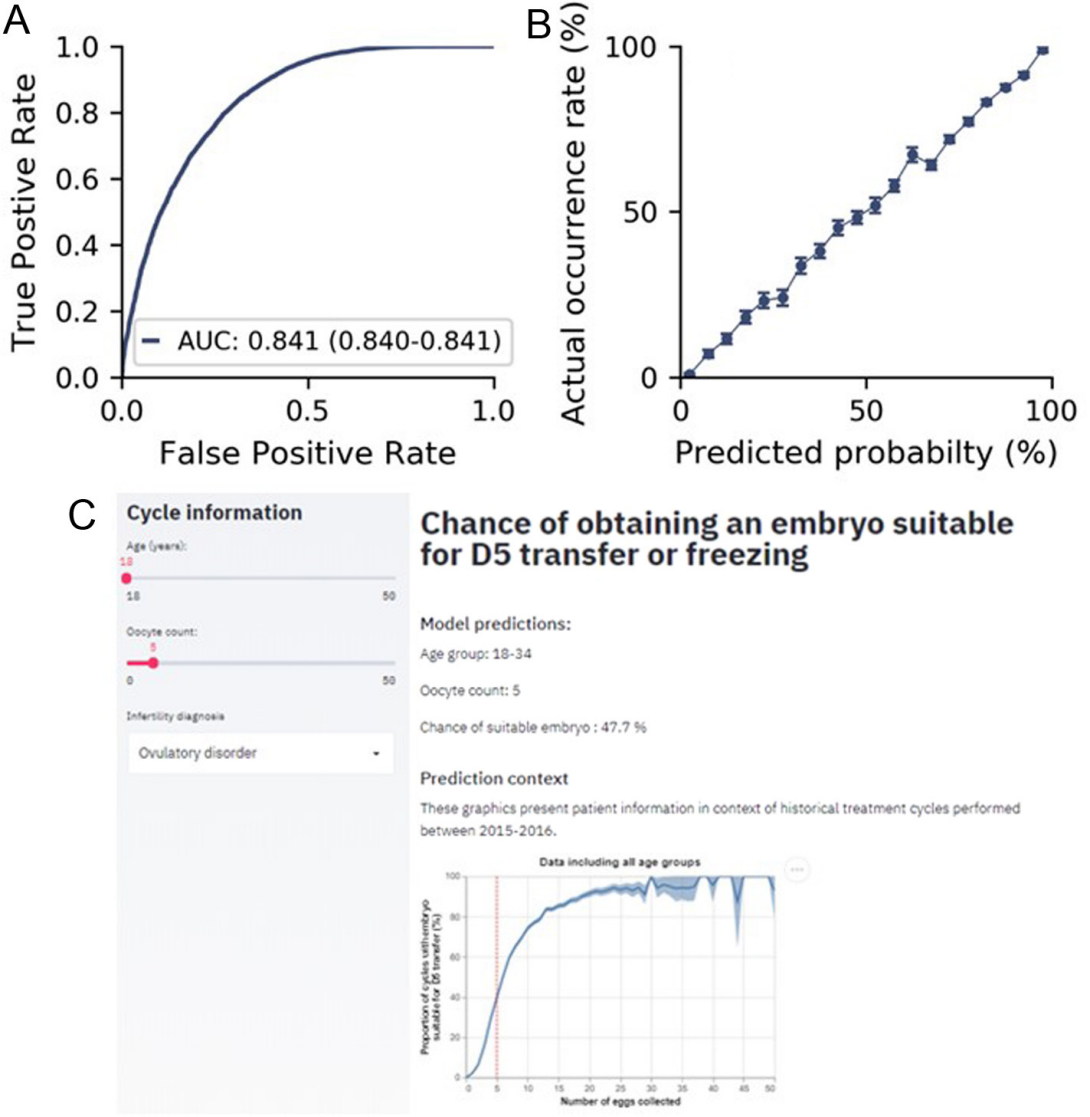
Performance statistics (A), calibration curve (B) and screenshot from web app for prediction of the chance of a day 5 embryo for transfer or freezing based on age, number of eggs collected and infertility diagnosis.

In this cohort, 56.53% of cycles yielded ≥1 embryo suitable for D5 transfer or cryopreservation and 43.47% did not. The number of oocytes retrieved is the primary predictor for any treatment cycle, but the patient’s age is an additional independent predictive factor. After optimizing the performance, a three-feature model (age, number of oocytes collected, and infertility diagnosis) predicted with high accuracy (AUROC of 0.841 (0.840–0.841) on the hold-out test set if a patient will obtain ≥1 suitable embryo. To demonstrate this model in practice, we have constructed a predictive tool to assist clinicians (https://fertility-predict.herokuapp.com/) (Fig. 1).

## Limitations

There were low numbers of treatment cycles for age ≥ 43 years so predictive confidence was lower in this group, necessitating a disclaimer on the web app. The model will predict pessimistically for patients with a poor prognosis as they are more likely to have an early stage transfer, particularly during the time period studied. The outcome may be confounded by different embryo cultures and transfer policies. Overall, model accuracy could be improved by including male partner parameters or previous outcome data.

## Discussion

The advantage of models like the one presented here is that they allow information held in historical fertility treatment records to be distilled and presented in a manageable way to clinicians, allowing informed clinical support. Clinicians can consider sharing the tool results with patients or use it to guide expectation-setting conversations. As records grow and information recorded about previous cycles becomes more detailed, predictions from these algorithms will increase in quality.

## Declaration of interest

The authors declare that there are no competing interests. Prof. Y Cheong is an Associate Editor of Reproduction and Fertility. Prof. Y Cheong was not involved in the review or editorial process for this paper, on which she is listed as an author.

## Funding

This work did not receive any specific grant from any funding agency in the public, commercial or not-for-profit sector.

## Data availability

The anonymised HFEA registry for all cycles performed in the UK in 2015–2016 is available to researchers online at no cost (HFEA 2018).

## Code availability

All code used to construct this predictive model have been publicly released on GitHub (Chmiel 2020).

## Author contribution statement

I Robertson envisaged the study, contributed to machine-learning modelling and led manuscript writing. F P Chmiel led machine-learning model development, created model code base, developed web application and contributed to manuscript writing. Y Cheong provided clinical insight, contributed to manuscript writing and supervised the project. All authors approved the final version to be published.
